# Poverty, redistribution, and the middle class: redistribution via probability distributions vs. redistribution via the linear income tax system

**DOI:** 10.3389/fsoc.2023.1334925

**Published:** 2024-02-02

**Authors:** Guillermina Jasso

**Affiliations:** Department of Sociology, New York University, New York, NY, United States

**Keywords:** poverty, redistribution, middle class, lognormal distribution, Pareto distribution, linear income tax system, inequality, income fairness and tax fairness

## Abstract

It has been known for a long time that (1) when graphs of income amount on income relative rank for two income distributions intersect twice, three “transfer groups” are generated, with the poorest and richest both gaining under the same alternative income distribution and the middle group losing; and (2) the linear income tax system satisfies three fundamental principles of tax justice, namely, that as pretax income increases, three quantities should also increase—posttax income, tax amount, and tax rate. This paper links those two ideas, suggesting that the linear income tax system may be the natural and most effective way to guard against poverty reduction policies which, while helping the poorest, as urged by Rawls, may harm the middle, contributing to the weakening of the middle class, thought at least since Aristotle to be the backbone of society. This paper illustrates the two approaches with one initial distribution and three alternative final distributions, contrasting their minimum, median, proportion below the mean, and inequality. It also shows how to guard the linear income tax system against violating the tax amount principle of tax fairness when there is an injection of resources (e.g., from deficit spending or oil revenues) and how to empirically estimate the parameters (e.g., the marginal tax rate) of the linear income system that the population will regard as fair.

## 1 Introduction

It has been known for a long time that (1) when graphs of income amount on income relative rank for two income distributions intersect twice, three “transfer groups” are generated, with the poorest and richest both gaining under the same alternative income distribution and the middle group losing (Schutz, 1951; Budd, 1970; Budd and Seiders, 1971; Jasso, 1983); and (2) the linear income tax system satisfies three fundamental principles of tax justice, namely, that as pretax income increases, three quantities should also increase—posttax income, tax amount, and tax rate (Fei, 1981; Musgrave, 1959, 1963; Intriligator, 1979; King, 1983; Seidl, 2007a,b; Jasso and Wegener, 2022). This paper links those two ideas, suggesting that the linear income tax system may be the natural and most effective way to guard against poverty reduction policies which, while helping the poorest, as urged by Rawls (1971), may harm the middle, contributing to the weakening of the middle class, thought at least since Aristotle (1952) (*Politics*, Book IV) to be the backbone of society, the “solid core” (Blau, 1964, 296-297).^1^

The study of poverty—how to define it, how to measure it, and how to alleviate it—has a rich and long history. Plato (c.428–348/7 BCE), concerned that societies suffer from both poverty and wealth, has the Athenian Stranger say (Plato, 1952, *Laws*, Book V):

[T]here should exist among the citizens neither extreme poverty, nor, again, excess of wealth, for both are productive of both these evils [faction and distraction]…[The minimum lot assigned in the beginning] ought to be preserved, and no ruler, nor anyone else who aspires after a reputation for virtue, will allow the lot to be impaired in any case… [The law] will permit a man to acquire double or triple, or as much as four times the amount of this [minimum lot]. But if a person have yet greater riches, whether he has found them, or they have been given to him, or he has made them in business, or has acquired by any stroke of fortune that which is in excess of the measure, if he give back the surplus to the state, and to the Gods who are the patrons of the state, he shall suffer no penalty or loss of reputation.

St. John Chrysostom (c.347–407 CE), in the *Homily 66.3*, *On the Gospel of Matthew* (Chrysostom, 1860, c. 386–407), estimated the proportions rich and poor in the prosperous city of Antioch at one-tenth each.^2^ Other highlights in the voluminous literature spanning philosophy, social sciences, humanities, and public policy include Fisher (1992), Friedman (1965), Fuchs (1967), Himmelfarb (1984), Orshansky (1965), Rawls (1971), Smith (1976a,b), and Vives (1947, 1520–1540), together with single-country and cross-country scholarly research on all aspects of poverty, as well as reports by the major international organizations (e.g., the United Nations and the World Bank) and non-profit “think tanks.”^3^

This paper first explores the two originating ideas and then links them. Of course, inequality is an active partner in the fate of the poor and the middle class. Indeed, even earlier than Plato, Confucius (c. 551–c. 479 BCE) taught that the underlying driver of social ills is inequality (An, 2021). Accordingly, inequality is never far from our discussion.

The present work may come at a propitious time, as the world prepares to celebrate the 500th anniversary of the classic and probably earliest book on poverty reduction, *De Subventione Pauperum* (*On the Relief of the Poor*), written in 1526 by Juan Luis Vives (1493–1540) for the Senate of Bruges.

## 2 Distribution and redistribution of income via probability distributions

We begin with two iconic probability distributions widely used in the study of wages, earnings, income, wealth, and other money variables (for convenience, termed “income”)—indeed, “the two classical size distributions” (Kleiber and Kotz, 2003, 126, 238): the lognormal and the Pareto.^4^ They are continuous univariate two-parameter distributions defined on positive numbers. The two parameters are a location parameter (such as the mean) and a shape parameter, which governs all measures of relative inequality and is thus called a general inequality parameter, denoted *c* (Jasso and Kotz, 2008). Both distributions permit incomes to range to infinity. However, they differ in their behavior at the bottom; while the lognormal goes to zero (from the right), the Pareto has a minimum positive amount. Thus, the lognormal is a reasonable representation of initial income and the Pareto for final income, the Pareto’s minimum income representing the social safety net. There is a large literature on the two distributions, to which Kleiber and Kotz (2003) provide valuable and succinct introduction, together with an introduction to size distributions in general and biographies of the major originators and contributors.^5^

For studying poverty and the middle class, important dimensions of income distributions include the minimum, the median, and the proportion below the arithmetic mean, as well as overall inequality and inequality within selected regions. For overall inequality, we report three measures: the widely used Gini coefficient (Kleiber and Kotz, 2003, 30) and two members of the class of generalized entropy inequality measures (Cowell and Kuga, 1981), the Theil MLD (Theil, 1967, 125–127) and the Atkinson (1970, 1975) measure (here called ATK) that arises when the inequality aversiveness parameter in the Atkinson family approaches one. All have excellent properties and an array of partisans. For example, Shorrocks (1980, p. 625) views the MLD as the “most satisfactory of the decomposable measures,” and Cowell and Flachaire (2023, p. 23) observe that the MLD “has all of the attractive properties of the Gini coefficient” and also “estimates variations in inequality more accurately” than the Gini. We also report the share held by the top 1%, at least since 2011 an iconic marker of inequality (Atkinson et al., 2011; Stiglitz, 2011; Jasso, 2020). In continuous, univariate two-parameter distributions, the Gini, Atkinson, Theil, and top share measures are monotonic functions of the general inequality parameter. For measuring inequality among the poor, following Parolin et al. (2023), we use the ratio of the 15th percentile to the 5th percentile. Formulas for all these measures in both mathematically specified and observed distributions are widely available (e.g., Jasso, 1980, 2018, 2020; Jasso and Kotz, 2008, p. 38–39).^6^

For visualization, we rely on graphs of income relative amount on income relative rank, formally, on graphs of the quantile function (QF) for income relative amount.^7^ If income is equally distributed (the benchmark case), the graph is a horizontal line at one (the average of the relative amounts). For unequal distributions, the graph increases as relative rank increases. As Pen (1971) put it, the QF for income distribution is like a parade that begins with dwarfs and ends with giants. Importantly, the flatter the curve, the lower the inequality, and there may be regions of greater or lesser flatness.^8^

Even more important, for present purposes, is that the QF signals not only the magnitude of inequality but also, in comparisons of two or more distributions, the exact cutoff points identifying who gains and who loses in a switch from one distribution to another (Schutz, 1951; Budd, 1970; Budd and Seiders, 1971; Jasso, 1983). When the alternatives pertain to absolute income, it is possible that everyone gains or everyone loses. However, when the alternatives pertain to relative income, there are always winners and losers; the QF graphs cross at least once, providing information about both the relative ranks and the relative income amounts at the boundaries of the “transfer groups.” Individuals are better off in the distribution whose QF graph gives them the higher vertical placement. When two QF graphs of relative income cross once, all transfers occur in one direction. Thus, there are two transfer groups, and the point of intersection shows whether the leftmost or the rightmost group is in the majority. When two QF graphs of relative income cross twice, transfers occur in both directions. In this case, there are three transfer groups, with the poorest and the richest gaining under the same alternative and the middle group losing. The three transfer groups can manifest any configuration of location and size, such as the middle group being the majority or the minority.^9^^,^ ^10^

### 2.1 Representing the initial income distribution by the lognormal

The lognormal (Aitchison and Brown, 1957; Kleiber and Kotz, 2003, p.107–145), whose application to economic variables began with the pioneering work of Gibrat (1931), is the classical distribution for the case where incomes can be both very low (going to zero) and very high (going to infinity). It would not be a good model for the initial income distribution in eras when pay schemes have rigid minimums or, as in Elizabethan England, rigid maximums (McArthur, 1900). However, it is not unreasonable as an early step in representing the initial distribution in contexts where it is possible to earn both very little and very much.^11^

**Figure 1:**
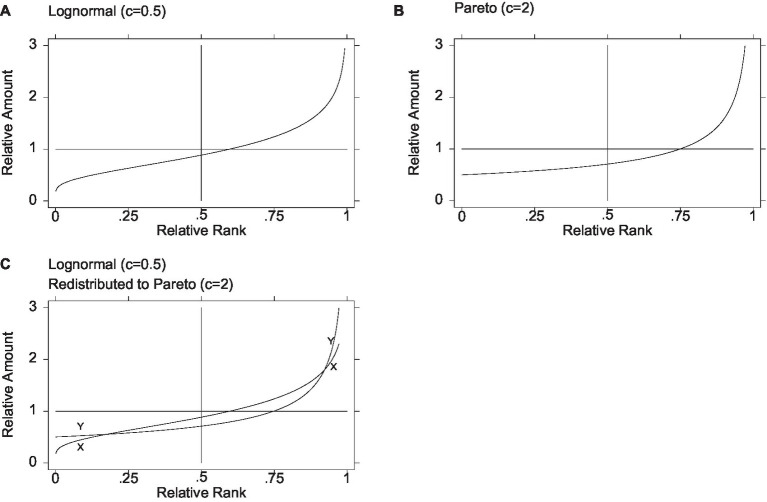
Redistribution from lognormal to Pareto. The poor and rich are better off, and the middle is worse off. The initial Lognormal distribution denoted *X* and the final Pareto distribution denoted *Y*. Figure 1C is based on Figure 3(a) in Jasso (2015, p. 889).

Figure 1A shows the graph of the relative amount on relative rank for one member of the lognormal family, with general inequality parameter *c* equal to 0.5. The grid features a vertical line at the median and a horizontal line at the mean (equal to one in a distribution of relative amounts, as noted above). The graph enables visualization of the relative income corresponding to each relative rank, for example, at the 25th percentile, the median, or the mean. The median is approximately 0.882, and the mean occurs at approximately the relative rank of 0.599, so approximately 59.9% have incomes below the mean. As for overall inequality, the values of the Gini, MLD, and ATK measures are 0.276, 0.125, and 0.118, respectively. The share held by the top 1% is 3.39%. Moreover, the graph makes it possible to gauge the amount of inequality in selected regions of the distribution; for example, following Parolin et al. (2023), inequality among the poor can be assessed by taking the ratio of the 15th to the 5th percentile. In the lognormal depicted in Figure 1A, the 5th and 15th percentiles are 0.388 and 0.526, yielding a P15/P5 ratio of 1.356. Similarly, eyeballing flatness in the curve, it is clear that inequality is much greater among the top 25% than among the bottom 25%.

### 2.2 Representing the final income distribution by the Pareto

The Pareto (Kleiber and Kotz, 2003, 59–106), pioneered by Pareto (1895), accommodates a distribution where incomes have a positive minimum and can also be very high—going to infinity. It is a reasonable model for the final income distribution in contexts where redistribution assures a social safety net but allows high earners to retain large amounts.

Figure 1B shows the graph of relative amount on relative rank for the member of the Pareto family with general inequality parameter *c* equal to 2. As with the lognormal in Figure 1A, the grid features a vertical line at the median and a horizontal line at the mean, and the graph enables visualization of the relative income corresponding to each relative rank. The minimum is half the mean, the median is ((√2)/2≈) 0.707, and the mean occurs at approximately the 75th percentile. As for overall inequality, values of the Gini, MLD, and ATK measures are 0.333, 0.193, and 0.176, respectively. The share held by the top 1% is 10%—higher than in the lognormal (*c* = 0.5), highlighting the divergent paths that inequality and poverty can take. Meanwhile, the 5th and 15th percentiles are 0.513 and 0.542, so that, following Parolin et al. (2023), the P15/P5 ratio registers 1.057, suggesting less inequality among the poor than in the lognormal. Similarly, the curve is much flatter among the bottom half than among the top half, suggesting far less inequality among the bottom half than among the top half.

### 2.3 Redistribution from the lognormal (*c* = 0.5) to the Pareto (*c* = 2)

Suppose now that a society with an initial distribution approximated by the lognormal (*c* = 0.5) decides to redistribute to a Pareto (*c* = 2), perhaps overlooking the facts that in this Pareto 75% of the incomes are below the mean and inequality is greater (e.g., a Gini of 0.333 vs. 0.276 and a top 1% share of 10% vs. 3.39%), so compelling is the other fact that the lowest income cannot go below half the mean. Figure 1C illustrates the result: three transfer groups, with the leftmost and the rightmost gaining and the middle group losing.^12^ In redistributions of relative income from a lognormal to a Pareto, there are three transfer groups—the bottom and top are always better off in the Pareto and the middle in the lognormal—but the transfer groups may differ markedly in their size and boundaries. In the case of the two distributions used in this illustration, the lognormal and the Pareto curves, denoted X and Y, respectively, cross at approximately the 17th and 92nd percentiles, so that a large middle majority of some 75% becomes worse off, while the poorest 17% and the richest 8% become better off. Put differently, persons immediately above the 17th percentile who might have expected to benefit from redistribution to the poor appear to be underwriting the advancement of persons not much poorer than themselves.^13^^,^^14^

The illustration in this section evokes contemporary discussions of the middle region of the income distribution losing ground (Stiglitz, 2015; Case and Deaton, 2020). Moreover, it prompts a search for a possibly better way to achieve poverty reduction.

## 3 Distribution of income via probability distribution, redistribution of income via the linear income tax system

The linear income tax system has been widely studied (Fei, 1981; Musgrave, 1959, 1963; Intriligator, 1979; King, 1983; Seidl, 2007a,b; Jasso and Wegener, 2022), and its properties and simplicity make it an appealing option. It satisfies the three principles of tax justice, namely, that as pretax income increases, so do final income, tax amount, and tax rate. The linear income tax system begins with a simple equation in which posttax income *y* is a linear function of pretax income *x*. The slope *b* represents the fraction of pretax income kept by the taxpayer, not counting the intercept *a*. When *a* is positive, it represents the minimum posttax income, known as the demogrant or social component, and, in Islamic law, the Nisab (Seidl, 2007a,b; Jasso and Wegener, 2022). If *a* is zero, the linear tax system reduces to a flat tax, which violates the third principle of tax justice, failing progressivity. Formally, the just linear tax system is written as:


y=a+bx,

a>0,0<b<1

### 3.1 Properties of the just linear tax system

Exploring the just linear tax system in a population of taxpayers leads to three useful further properties (Jasso and Wegener, 2022). First, besides the intercept *a* and slope *b*, there is a third parameter, the level of extraction/injection of resources, represented by the ratio of the average posttax income to the average pretax income and denoted *k*. Resources may be extracted to fund the government, for security, etc. Conversely, the injection of resources may reflect colonial revenues, oil revenues, deficit spending, or economic growth.^15^ Importantly, when pretax income and posttax income are expressed as relative amounts, the parameter *k* equals one.

Second, embedded in the just linear tax system is a standard form of the intercept and slope, denoted by asterisks and called the signature system. This standard pair—*viz.*, a* and b*—occurs naturally when pretax income and posttax income are expressed as relative amounts and has the property that the two parameters sum to one. Moreover, because a* represents the just relative minimum final income and 1−b* is the standard form of the marginal tax rate, it follows that a* represents both the just relative minimum final income and the marginal tax rate. Thus, “increasing the relative minimum is the same as increasing the marginal tax rate” (Jasso and Wegener, 2022, p.210), and the fates of the poor and rich are intertwined.

Third, it turns out that the second principle of justice—that as pretax income increases, the tax amount should also increase—is violated if the injection of resources grows to the point that *k* reaches or exceeds 1/b*. Thus, for given signature parameters, it is possible to know *a priori* how much injection of resources can be tolerated without violating the second principle (Jasso and Wegener, 2022, p. 213, Table 4).

### 3.2 Redistribution from the lognormal (*c* = 0.5) via the just linear tax system

Suppose now that a society with an initial distribution approximated by the same lognormal (*c* = 0.5) as in Section 2 decides to redistribute via the linear tax system. Two tax systems are considered, specified by their signature standard parameters. The first signature set has a* of 0.225 and b* of 0.775. This is the signature parameter set estimated empirically for a probability sample of German earners in 2009, based on their responses to questions asked in the German SocioEconomic Panel (GSOEP) about their current actual pretax income and their idea of the just posttax income for themselves (Jasso and Wegener, 2022). The second signature set has a* of 0.3 and b* of 0.7. Of course, the society could specify any other signature set, cognizant that a* represents the standard form of both the relative minimum income and the marginal tax rate, or it could estimate empirically its population’s ideas about the just system.^16^

**Figure 2:**
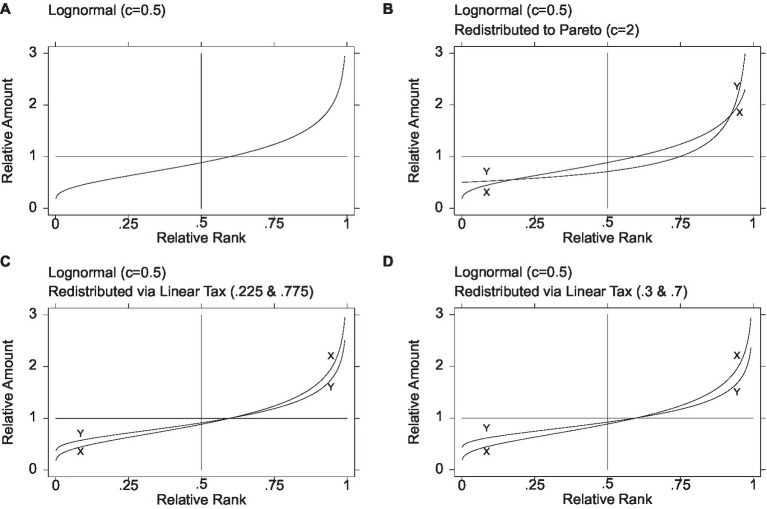
Redistribution from lognormal via linear tax. Leftmost better off, rightmost worse off (plus contrast with redistribution to Pareto). The initial lognormal distribution denoted *X* and the final distributions denoted *Y*. Figure 1B is based on Figure 3(a) in Jasso (2015, 889)

Figure 2C depicts the graphs of relative amount on relative rank for the pretax distribution (lognormal of *c* = 0.5), denoted *X*, and the posttax distribution obtained from the 0.225 to 0.775 tax system, denoted *Y*. As shown, the two graphs intersect only once, at the mean of one and the relative rank of approximately 0.599. Thus, the leftmost 60% have gained from the redistribution, and the rightmost 40% have lost. The lowest income has increased from close to zero (in a population of a 1,000, approximately 18.8% of the mean) to 37.1% of the mean. The highest income has declined from 4.14% of the mean (again, in a population of a thousand) to 3.43% of the mean. Of course, in a population of millions, the figures at the extremes would differ. The three overall inequality measures—Gini, MLD, and ATK—register 0.213, 0.0713, and 0.0688, respectively, indicating a nontrivial decline in inequality. The share held by the top 1% is 2.76%. Finally, to gauge inequality among the poor, following Parolin et al. (2023), we obtain the 5th and 15th percentiles (0.525 and 0.632), yielding a P15/P5 ratio of 1.203 and suggesting less inequality among the poor than in the lognormal, where it is 1.356 (but more than in the Pareto, where it is 1.057, visible in the curve’s flatness).^17^

Now suppose that a combination of economic growth and deficit spending permits the average of the final income distribution to increase. How much could be used in redistribution without violating the second principle of tax justice? The answer is that *k* could increase to 1/b*, or approximately 1.29, for an injection of resources equal to 29% of the initial income distribution (Jasso and Wegener, 2022, p. 213, Table 4). If the society wanted to take *k* to 1.5, injecting resources equal to half the initial total income, then to safeguard the second principle of tax justice, the tax system parameters would have to change to 0.333–0.667 (Jasso and Wegener, 2022, p. 214, Table 5). That is, the standard relative minimum final income (and, identically, the standard marginal tax rate) would have to increase from 0.225 to 0.333, or approximately 33%.

Analyzing the 0.3–0.7 tax system exactly as above for the 0.225–0.775 system, Figure 2D depicts the graphs of relative amount on relative rank for the pretax distribution (lognormal of *c* = 0.5) and the posttax distribution obtained from the 0.3–0.7 tax system. As shown, the two graphs intersect only once, at the mean of one and the relative rank of approximately 0.599. This is exactly as in the 0.225–0.775 system, which is not surprising given that the tax system is a linear transformation. The lowest income has increased from close to zero (in a population of a thousand, approximately 18.8% of the mean) to 37% of the mean. The highest income has declined from 4.14% of the mean (again, in a population of a thousand) to 3.20% of the mean. Of course, in a population of millions, the figures at the extremes would differ. The three overall inequality measures—Gini, MLD, and ATK—register 0.192, 0.0579, and 0.0563, respectively, indicating a further decline in inequality. The share held by the top 1% is 2.59%. Finally, to gauge inequality among the poor, following Parolin et al. (2023), the 5th and 15th percentiles are 0.571 and 0.668, yielding a P15/P5 ratio of 1.169 and suggesting less inequality among the poor than in the lognormal (where it is 1.356) or via the 0.225–0.775 system (where it is 1.203) but more than in the Pareto (where it is 1.057).

As mentioned above, we now ask how much of a windfall (or deficit spending) could be used in redistribution without violating the second principle of tax justice. The answer is that *k* could increase to 1/b*, or approximately 1.429, for an injection of resources equal to 42.9% of the initial income distribution (Jasso and Wegener, 2022, p.213, Table 4). If the society wanted to take *k* to 1.5, injecting resources equal to half the initial total income, then, exactly as above, to safeguard the second principle of tax justice, the tax system parameters would have to change to 0.333–0.667 (Jasso and Wegener, 2022, p.214, Table 5). That is, the standard relative minimum final income (and, identically, the standard marginal tax rate) would have to increase from 0.3 to 0.333, or approximately 33%.

## 4 Contrasting the two approaches to redistribution and poverty reduction

Figure 2 makes it possible to visualize at a glance the original pretax distribution and the three posttax distributions achieved via a probability distribution and via the linear tax system. It is evident that while redistribution via the Pareto (Figure 2B) renders a middle region worse off, redistribution via the linear tax system (Figures 2C,D) does no harm to the middle. For further concreteness, Table 1 reports all the key measures reported above—the minimum, the median, the proportion below the mean, the three measures of overall inequality, the share held by the top 1%, and the P15/P5 measure of inequality among the poor.

**Table 1:** Key measures in the pretax and posttax distributions.

Key measures	Pretax distribution	Posttax distributions		
	Lognormal (*c* = 0.5)	Redistribution to Pareto (*c* = 2)	Redistribution via Linear Tax (0.225–0.775)	Redistribution via Linear Tax (0.3–0.7)
Minimum	→0+	0.5	0.371	0.432
Median	0.882	0.707	0.909	0.918
Proportion below Mean	0.599	0.75	0.599	0.599
Gini Coefficient	0.276	0.333	0.213	0.192
Theil MLD	0.125	0.193	0.0713	0.0579
Atkinson inequality	0.118	0.176	0.0688	0.0563
Share held by Top 1%	3.39%	10%	2.76%	2.59%
P15/P5	1.356	1.057	1.203	1.169

In this illustration, the pretax distribution is modeled by the lognormal (*c* = 0.5), and the three posttax distributions are modeled by the Pareto (*c* = 2) and two just linear tax distributions with signature standard parameters of 0.225–0.775 and 0.3–0.7. The lognormal has no safety net and no maximum; the Pareto has a safety net but, like the lognormal, no maximum. In all four distributions, income is represented by relative amounts, viz., the absolute amount divided by the arithmetic mean; thus, the arithmetic mean in these relative-amount distributions equals one. The P15/P5 ratio measures the inequality among the poor (Parolin et al., 2023). Values for the key measures in the lognormal and Pareto distributions are approximated via the corresponding formulas in mathematically specified distributions, and values in the posttax distributions obtained via the linear tax are approximated by the corresponding numerical formulas in observed distributions (Jasso, 2018, 2020; Jasso and Kotz, 2008, 36–39).

It is evident from both Table 1 and Figure 2 that the Pareto option provides the highest minimum income. We may wonder whether even the most committed Rawlsian would hesitate before choosing it, knowing that it harms the middle, has the lowest median, has the largest proportion below the mean, and has the highest overall inequality. The differences in this illustration are not subtle. The Pareto’s minimum income of half the mean is larger than the two tax systems’ minimums of 0.371 and 0.432 of the mean. The Pareto’s median of 0.707 is smaller by over 20 percentage points than the two tax systems’ medians of 0.909 and 0.918. The Pareto’s 75% proportion below the mean exceeds by 15 percentage points the proportion below the mean in the two tax systems’ redistribution. Further, by all the measures of overall inequality, the Pareto’s overall inequality is the largest—for example, a Gini coefficient of 0.333, over 10 percentage points greater than the two tax systems’ Ginis of 0.213 and 0.192. Finally, the share held by the top 1% is over three times greater in the Pareto than in the two tax systems (10% vs. 2.76% and 2.59%). In the Pareto’s defense, however, the P15/P5 ratio registers the lowest magnitude of inequality among the poor—1.057 vs. 1.203 and 1.169.

Of course, this illustration is based on specific members of the lognormal and Pareto distributional families and specific signature parameters of the linear tax system. Exact results will differ by the exact combinations.^18^ What might be some general conclusions? First, the iconic Pareto is no panacea, given its harm to the middle. Second, redistributions via members of lower inequality from the same distributional family will not harm the poor, as they generate only two transfer groups, and thus it would seem useful to explore “shifted” or “generalized” forms of the lognormal as approaches to both distribution and redistribution. Third, it would also seem useful to explore other distributional families, such as the shifted exponential, the shifted gamma, and the quadratic with its beautiful symmetry.^19^

## 5 Discussion

This paper combined two well-known ideas—(1) redistribution from one to another distribution can yield three transfer groups such that the poorest and richest gain and the middle loses and (2) the linear income tax system satisfies three basic principles of fairness, namely, that as initial income increases, so do final income, tax amount, and tax rate, suggesting that the linear income tax system may be the natural and most effective way to guard against poverty reduction policies which, while helping the poorest, harm the middle.

Further work might examine, more deeply and both theoretically and empirically, the links between poverty, redistribution, and the middle class, on the one hand, and inequality and both income fairness and tax fairness, on the other hand.

With respect to income fairness, there are at least three links ripe for further study. First, justice theory provides a decomposition of overall injustice (the average of the individual-specific justice evaluations) into injustice due to poverty and injustice due to inequality (Jasso, 1999, 2023), enabling new empirical research on the question of whether injustice is poverty-led or inequality-led in approximately 30 European countries (with longitudinal information available as well for Germany, as noted in text footnote 16). Second, justice theory provides a further decomposition of overall injustice based on the MLD:


$$E\left(J\right)=\ln\left[E\left(X\right)\right]-\ln\left[E\left(X^{*}\right)\right]-\text{MLD}\left(X\right)+\text{MLD}\left(X^{*}\right)$$

where *X* denotes actual income and X* denotes just income. This new decomposition makes it possible to look closely at what the societal data reveal about how individuals form their ideas of the just reward for self, in particular, whether individuals are converging on an idea of the just reward and whether that idea is close to the average actual reward. Third, both the ATK and MLD inequality measures have exact links to a special case of overall injustice (in which subjective ideas of just income coalesce around the average). In fact, in this special case, overall injustice equals the negative of the MLD (Jasso, 1999, 2023; Cowell, 2011). Importantly, the new European data permit estimation for both pretax income and posttax income.

With respect to tax fairness, the new data permit approximation of the specific linear tax scheme regarded as fair in almost 30 European countries in Round 9 of the European Social Survey, building on the German study discussed above. These data will enable comparison for the first time of just linear tax schedules across a set of countries.

With respect to the link between income fairness and tax fairness, the stage is set for both new theoretical work and new empirical work linking the principles of income justice (such as need, merit, and equality and including both principles of microjustice and principles of macrojustice) and the principles of tax justice (pertaining to the final income, the tax amount, and the tax rate, as discussed in this paper). Note that if individuals or societies judge that the initial distribution is unjust, they will not want to use it as the base for redistribution, but instead will first modify it to achieve a just pretax distribution before continuing to the redistribution to achieve a just posttax distribution. The European Social Survey and the GSOEP, with their data on both just pretax income and just posttax income, will be invaluable for studying this more complicated scenario.

Finally, consider that there are two types of inequality—inequality between persons (the inequality measured by the Gini, Theil MLD, and Atkinson measures) and inequality between subgroups (the inequality measured by ratios and gaps)—as known for a long time [at least since Jencks et al. (1972) and later systematized in Jasso and Kotz (2008)]. To this point, all the inequality considered in this paper has been inequality between persons. Yet subgroup inequality—inequality between subgroups defined by qualitative characteristics, such as race, gender, ethnicity, nativity, citizenship, language, religion, and so on—may have special links to poverty, poverty reduction, and the middle class, and these warrant careful study.

Consider gender. Plato and Confucius, near contemporaries in areas of the world that had apparently not yet met, both thought inequality was the source of societal ills. While Plato (*Meno*; *Republic*, Book V) found in nature no bar to full gender equality (Jasso, 2011), his pupil Aristotle and Confucius found it straightforward to assign bondage to half the world. Did Gregory the Great (1849, 540–604) think of gender when he observed in (1849) *Moralia in Job* xxi, “Where there is no sin, there is no inequality”? The retorts would come, but sporadically and not in torrents: Jerome (347–419/420), pleading with a friend’s daughter not to marry (Jerome, 1845–1846, Letter XXII, to Eustochium, written in A.D. 384); Anselm (1033/4-1109), addressing saints and gods as Mother (Fortin, 2017); and Vives (1947) writing “On the Duties of a Christian Husband”. Today, we may ask whether poverty or harm to the middle class is more tolerated in one gender than another. This is a topic ready for study all over the world. Of course, the same questions can be asked about other subgroup categorizations beyond gender—race, ethnicity, nativity, citizenship, language, religion, and so on.

## Author contributions

GJ: Conceptualization, Formal analysis, Visualization, Writing – original draft, Writing – review & editing.

## Footnotes

^1^The term “tax” includes both taxes and transfers; “tax” can be positive (tax paid) or negative (a subsidy). The terms “pretax” and posttax” are used interchangeably with “initial” and “final,” respectively.

^2^Chrysostom apparently defines rich and poor by specified income amounts and estimates the proportion in each class, providing an early example of one of the two major approaches to class structure, the other being to define classes by specified proportions of the population and estimate the relative share of income held by each class (Jasso, 1983, 284).

^3^For example, for a succinct overview of scholarly research on poverty, family factors, measurement equivalence, and other pertinent matters in the United States and Europe, see Davidov et al. (2014), Edin and Nelson (2013), Edin and Shaefer (2015), Parolin et al. (2023), Reinl et al. (2023), Sosu and Schmidt (2017), and the references cited therein. Of course, poverty plays an important part in literature, art, even music, as in Monteverdi’s (1567-1643), Bach’s (1685-1750), and others’ settings of Mary’s lines, *esurientes implevit bonis, et divites dimisit inanes*, “The hungry he has filled with good things, and the rich he has sent away empty” (Magnificat, Luke 1:53).

^4^Because all income distributions are subject to the same underlying mathematical relations of probability distributions, this approach makes it possible to explore the relation between poverty and the middle class with some generality.

^5^The shape parameter appears by different names in different distributions. For example, in the Pareto, it is called Pareto’s constant and is usually denoted by α. The Pareto parameter is sometimes expressed not as *c* (=α) but as an inverted Pareto coefficient denoted β and equal to *c*/(*c*−1). This usage has two useful properties: (1) inequality increases as β increases; and (2) the average of the top subgroup is equal to β times the *x* value at the percent split *p*. See, for example, Alvaredo (2011, p. 275) and Atkinson et al. (2011, p. 13–14).

^6^The Theil MLD and the Atkinson measure both depend on ratios of the geometric mean and the arithmetic mean and are thus algebraic transformations of each other: ATK = 1 – exp(–MLD) and MLD = −ln(–ATK). Indeed, given that ATK sits on the unit interval but the MLD ranges beyond one, the Atkinson measure may be regarded as a “normalization” of the MLD (Cowell, 2011, p.186; Jasso, 2023, p. 312–313).

^7^Relative income is income divided by the arithmetic mean. If the incomes are arranged in ascending order, their absolute ranks range from 1 to the group or population size *N*, and relative rank is absolute rank divided by *N* + 1. Formally, graphs of (relative) income amount on income relative rank are graphs of the quantile function, one of the three main associated functions of probability distributions, also known as the inverse distribution function, the percent point function, and, when used for income or other money variables, Pen’s Parade (Pen, 1971; Cowell, 1977, 2011; Jasso, 1983; Atkinson, 2017; U.S. National Institute of Standards and Technology, 2018).

^8^Among the three main associated functions of probability distributions, the most basic is the cumulative distribution function (CDF), the best known is the probability density function (PDF), and perhaps the most useful in studies of income inequality is the quantile function. The three are related to each other in specified ways (Jasso, 1983, p.276–277). For example, the QF is the inverse of the CDF (hence one of its alternate names), and in continuous distributions the PDF is the first derivative of the CDF. For further detail on the QF and its relation to the other associated functions of probability distributions, together with formulas and graphs, see the Gallery of Distributions in the online *eHandbook of Statistical Methods* (also known as the *Engineering Statistics Handbook*) published by U.S. National Institute of Standards and Technology (2018); the one-volume handbook by Forbes et al. (2011); and the comprehensive compendia originated by Johnson and Kotz (1969-1972) and continued with Balakrishnan and Kemp (e.g., Johnson et al., 1994–1995, 2005). Formulas and graphs for the basic associated functions of the lognormal and Pareto are widely available, for example, in the sources above and Jasso and Kotz (2008, 36-37).

^9^As shown, the QF is useful not only for studying the income structure and how it changes but also, when individuals retain their ranks, how individuals become better-off and worse-off. Thus, two elements crucial to understanding income distribution and discussed by Marx (1818–1883) are important in this article—relative amounts (Marx, 1968) and rank invariance (Marx, 1964).

^10^We note in passing that the literature contains important links between the QF and inequality and between the QF and the Lorenz curve. For example, two QFs of relative income intersect one more time than the corresponding Lorenz curves, so that the canonical case of nonintersecting Lorenz curves is equivalent to the case of QFs intersecting once—and these indicate that the two distributions can be unambiguously ranked on their degree of inequality. Further pertinent literature includes Arnold and Sarabia (2018), Atkinson (1970, 1975, 2017), Budd (1970), Budd and Seiders (1971), Cowell (1977, 2011), Fields and Fei (1978), Jasso (1983, 2020), Kleiber and Kotz (2003), and Schutz (1951).

^11^Notable recent developments include (1) work by Battistin et al. (2009) noting that the lognormal’s fit is better for consumption than for income and that “the logic of Gibrat’s law applies not to total income, but to permanent income and to marginal utility” and (2) work by Venkatasubramanian (2017) and Venkatasubramanian et al. (2015) showing that self-organizing free-market dynamics reach an equilibrium state with an emergent equilibrium distribution which turns out to be lognormal.

^12^The three transfer groups visible in Figure 1C emerge from the two intersections of the distributions’ quantile functions. Equivalently, the two distributions’ Lorenz curves intersect once. Thus, this situation echoes Cowell’s (1977, 36, 49) insightful remarks that “there has been a redistribution away from middle-income receivers to the top *and* to the bottom” and that the “Lorenz curves that we wish to compare often intersect.”

^13^To illustrate the variability in size and boundaries of the transfer groups, if the initial income distribution were a lognormal (*c* = 0.6) rather than the lognormal (*c* = 0.5) in Figure 1, redistribution to the same Pareto (*c* = 2) in Figure 1 would lead to a smaller middle region of some 67% extending from the 28th percentile to the 95th percentile becoming worse-off, and if the initial income distribution were a lognormal (*c* = 1), redistribution to the same Pareto (*c* = 2) would lead to an even smaller “middle” region of some 38% extending from the 61st percentile to the 99.76th percentile becoming worse-off (Jasso, 1983, 291–294). Moreover, if the initial income distribution were a lognormal (*c* = 0.82), redistribution to the same Pareto (*c* = 2) would lead to the case in which the majority comprises the bottom 49% and the top 1.3%—and hence does not include the median person (Jasso, 2015, 888–889). Always, however, the “poorest” (or leftmost) and the “richest” (or rightmost) share the same fate, their interests opposite the interests of the “middle” region. Finally, a transfer group may be so tiny that it would be discernible only in a very large population; for example, in a redistribution from a lognormal (*c* = 2) to a Pareto (*c* = 2), the point of intersection between the “middle” region and the “top” region occurs between 0.999999999 and 0.9999999999.

^14^Of course, the lognormal and Pareto pair are not the only distributions giving rise to three transfer groups. Other examples include a power function and Pareto pair (Jasso, 2015, 888–889). As well, observed empirical distributions also may intersect. For example, Budd (1970) and Weisskoff (1970) report that in the United States in the 1945–1965 period and in Mexico in the 1950–1963 period, a large bottom segment and a small top segment became worse-off in relative income, while the middle group became better-off.

^15^For a line of inquiry exploring the relations among growth, redistribution, and poverty, see Chenery et al. (1974).

^16^Empirical approximation of the signature system viewed as fair by a population is straightforward if data are available on each respondent’s own actual pretax income and own just posttax income (*viz.*, the posttax income regarded as just for self by the respondent); the procedure simply regresses just posttax income on actual pretax income, both in relative form (Jasso and Wegener, 2022). Such data have been available in Germany since 2009 and are now available for almost 30 countries in the European Social Survey, Round 9 (2018–2019).

^17^Figures 2A,B repeat Figures 1A,C enabling visual contrast of all three redistributions.

^18^Consider the focal distributions used in this paper to model pretax and posttax income, the lognormal (*c* = 0.5) and the Pareto (*c* = 2), respectively. The reader will have noticed in the text and in Table 1 that by all three measures of overall inequality, the Pareto has greater inequality than the lognormal. But this is not universally the case. In fact, by the Gini measure, the Pareto (*c* = 2) is less unequal than any lognormal of *c* greater than 0.609, and by the Atkinson measure, it is less unequal than any lognormal of *c* greater than 0.622. Thus, there is a “zone of ambiguity” in ranking the Pareto (*c* = 2) and members of the lognormal family with *c* between 0.609 and 0.622 (Fields and Fei, 1978, p.315; Jasso, 1982, p.319–321).

^19^The four small “toy distributions” provided by Jasso (2018, 202) are based on the lognormal, Pareto, shifted exponential, and quadratic, and thus can be used to experiment with further options for redistribution. Note that the toy quadratic has a minimum income of half the mean and a maximum income of three times the mean, well within the maximum allowed by the Athenian Stranger in Plato’s *Laws* (quoted above).

## References

[ref1] Aitchison J. Brown J. A. C. (1957). The Lognormal Distribution. Cambridge, UK: Cambridge

[ref2] Alvaredo F. (2011). A note on the relationship between top income shares and the Gini coefficient. Economics letters 110:274–277.

[ref500] An W. (2021). Fear not scarcity but inequality, not poverty but instability. Sociol. Methods Res. 50, 939–943. doi: 10.1177/00491241211024295

[ref3] Aristotle (1952). The Works of Aristotle 2. Chicago: Britannica

[ref4] Arnold BC Sarabia JM (2018). Majorization and the Lorenz Order with Applications in Applied Mathematics and Economics. Cham, Switzerland: Springer

[ref5] Atkinson A. B. (1970). On the measurement of inequality. J. Econ. Theory 2, 244–263. doi: 10.1016/0022-0531(70)90039-6

[ref600] Atkinson A. B. (1975). The Economics of Inequality. London: Oxford

[ref6] Atkinson A. B. (2017). Pareto and the upper tail of the income distribution in the UK: 1799 to the present. Economica 84, 129–156. doi: 10.1111/ecca.12214

[ref7] Atkinson A. B. Piketty T. Saez E. (2011). Top incomes in the long run of history. J. Econ. Lit. 49, 3–71. doi: 10.1257/jel.49.1.3

[ref8] Battistin E. Blundell R. Lewbel A. (2009). Why is consumption more log normal than income? Gibrat’s Law Revisited. J. Polit. Econ. 117, 1140–1154. doi: 10.1086/648995

[ref9] Blau PM (1964). Exchange and Power in Social Life. New York: Wiley

[ref10] Budd E. C. (1970). Postwar changes in the size distribution of income in the U.S. Am. Econ. Rev. Pap. Proc. 60, 247–260.

[ref11] Budd E. C. Seiders D. F. (1971). The impact of inflation on the distribution of income and wealth. Am. Econ. Rev. Pap. Proc. 61, 128–138.

[ref12] Case A Deaton A (2020). Deaths of Despair and the Future of Capitalism. Princeton, NJ: Princeton University Press

[ref13] Chenery H Ahluwalia MS Bell C. L. G. Duloy JH Jolly R (1974). Redistribution With Growth. London, UK: Oxford.

[ref14] Chrysostom St. J. (1860) in Patrologiae Cursus Completus. Series Graeca. ed. Migne J. P., vol. 47–64. Paris: Imprimerie Catholique.

[ref15] Cowell FA (1977). Measuring Inequality: Techniques for the Social Sciences. New York: Wiley

[ref16] Cowell FA (2011). Measuring Inequality 3rd Edn. Oxford, UK: Oxford University Press

[ref17] Cowell F. A. Flachaire E. (2023). Inequality measurement and the rich: why inequality increased more than we thought. Rev. Income Wealth. doi: 10.1111/roiw.12638

[ref18] Cowell F. A. Kuga K. (1981). Additivity and the entropy concept: an axiomatic approach to inequality measurement. J. Econ. Theory 25, 131–143. doi: 10.1016/0022-0531(81)90020-X

[ref19] Davidov E. Meuleman B. Cieciuch J. Schmidt P. Billiet J. (2014). Measurement equivalence in cross-National Research. Annu. Rev. Sociol. 40, 55–75. doi: 10.1146/annurev-soc-071913-043137

[ref20] Edin K Nelson T. (2013). Doing the Best I Can: Fathering in America. Berkeley, CA: University of California Press

[ref21] Edin K. Shaefer H. L. (2015). Equity oriented fiscal programs. Econometrica 49, 869–881. doi: 10.15453/0191-5096.4003

[ref22] Fields G. S. Fei J. C. H. (1978). On inequality comparisons. Econometrica 46, 303–316. doi: 10.2307/1913902

[ref23] Fisher G. M. (1992). The development and history of the poverty thresholds. Soc. Secur. Bull. 55, 3–14. PMID: 1300640

[ref24] Forbes C Evans M Hastings N Peacock B (2011). Statistical distributions 4rth Edn. New York, NY: Wiley

[ref25] Fortin J. R. (2017). St. Anselm’s prayer to St Paul. St. Anselm J 13, 57–67.

[ref26] Friedman RD (1965). Poverty: Definition and perspective. American Enterprise Institute for Public Policy Research (Report). Washington, DC.

[ref27] Fuchs V. R. (1967). Redefining poverty and redistributing income. Public Interest 8, 88–95.

[ref28] Gibrat R (1931). Les Inegalites Economiques. Paris: Librairie du Recueil Sirey

[ref29] Gregory the Great (1849) in Patrologiae Cursus Completus. Series Latina. ed. Migne J. P. (Paris: Imprimerie Catholique), 75–79.

[ref30] Himmelfarb G (1984). The Idea of Poverty: England in the Early Industrial Age. New York, NY: Knopf

[ref31] Intriligator M. D. (1979). Income redistribution: a probabilistic approach. Am. Econ. Rev. 69, 97–105.

[ref32] Jasso G. (1980). A new theory of distributive justice. Am. Sociol. Rev. 45, 3–32. doi: 10.2307/2095239

[ref33] Jasso G. (1982). Measuring inequality by the ratio of the geometric mean to the arithmetic mean. Sociol. Methods Res. 10, 303–326. doi: 10.1177/0049124182010003004

[ref34] Jasso G. (1983). Using the inverse distribution function to compare income distributions and their inequality. Res. Soc. Stratific. Mobil. 2, 271–306.

[ref35] Jasso G. (1999). How much injustice is there in the world? Two new justice indexes. Am. Sociol. Rev. 64, 133–168. doi: 10.2307/2657282

[ref36] Jasso G. (2011). “Plato: seven sociological ideas for the happy life” in Sociological Insights of Great Thinkers: Sociology through Literature, Philosophy, and Science. eds. Edling C. Rydgren J. (Santa Barbara, CA: Praeger), 31–44.

[ref508] Jasso G. (2015). “Inequality analysis: Overview” in The International Encyclopedia of the Social and Behavioral Sciences, Second Edition. Volume 11. ed. Wright J. D. (London, UK: Elsevier), 885–893.

[ref37] Jasso G. (2018). What can you and I do to reduce income inequality? J. Math. Sociol. 42, 186–204. doi: 10.1080/0022250X.2017.1343826

[ref38] Jasso G. (2020). Anything Lorenz curves can do, top shares can do: assessing the TopBot family of inequality measures. Sociol. Methods Res. 49, 947–981. doi: 10.1177/0049124118769106

[ref39] Jasso G. (2023). Fifty years of justice research: seven signposts past and future. Soc. Justice Res 36, 305–324. doi: 10.1007/s11211-023-00419-5

[ref40] Jasso G. Kotz S. (2008). Two types of inequality: inequality between persons and inequality between subgroups. Sociol. Methods Res. 37, 31–74. doi: 10.1177/0049124108318971

[ref41] Jasso G. Wegener B. (2022). An empirically based just linear income tax system. J. Math. Sociol. 46, 195–225. doi: 10.1080/0022250X.2020.1859501

[ref42] Jencks C Smith M Acland H Bane MJ Cohen D Gintis H. (1972). Inequality: A Reassessment of the Effect of Family and Schooling in America. New York, NY: Basic Books

[ref43] Jerome S. (1845–1846) in Patrologiae Cursus Completus. Series Latina. ed. Migne J. P., vol. 22–30. Paris: Imprimerie Catholique.

[ref44] Johnson NL Kemp AW Kotz S (2005). Univariate Discrete Distributions. 3rd Edn. New York, NY: Wiley

[ref45] Johnson NL Kotz S. (1969–1972). Distributions in Statistics. Four volumes. New York, NY: Wiley

[ref46] Johnson NL Kotz S Balakrishnan N. (1994–1995). Continuous Univariate Distributions. Two volumes. New York, NY: Wiley

[ref47] King M. A. (1983). An index of inequality: with applications to horizontal equity and social mobility. Econometrica 51, 99–115. doi: 10.2307/1912250

[ref48] Kleiber C Kotz S (2003). Statistical Size Distributions in Economics and Actuarial Sciences. Hoboken, NJ: Wiley

[ref49] Marx K. (1964). The Eighteenth Brumaire of Louis Bonaparte. New York: International Publishers

[ref50] Marx K. (1968). “Wage labour and capital” in Karl Marx and Frederick Engels: Selected Works (New York: International Publishers), 74–97.

[ref51] McArthur E. A. (1900). The regulation of wages in the sixteenth century. Engl. Hist. Rev. XV, 445–455. doi: 10.1093/ehr/XV.LIX.445

[ref52] Musgrave R. A. (1959). The Theory of Public Finance. New York, NY: McGraw-Hill

[ref53] Musgrave R. A. (1963). Growth with equity. Am. Econ. Rev. 53, 323–333.

[ref54] Orshansky M. (1965). Counting the poor: another look at the poverty profile. Soc. Secur. Bull. 28, 3–29. 3068816

[ref55] Pareto V. (1895). La Legge della Domanda. Giorn. Degli Econom. 10, 59–68.

[ref56] Parolin Z. Desmond M. Wimer C. (2023). Inequality below the poverty line since 1967: the Rold of the U.S. welfare state. Am. Sociol. Rev. 88, 782–809. doi: 10.1177/00031224231194019

[ref57] Pen J. (1971). Income Distribution. London, UK: Allen Lane

[ref58] Plato (1952). The Dialogues of Plato. Translated by Benjamin Jowett. Chicago: Britannica

[ref59] Rawls J (1971). A Theory of Justice. Cambridge, MA: Harvard

[ref60] Reinl A. K. Seddig D. Dennison J. Davidov E. (2023). Basic human values and preferences for an EU-wide social benefit scheme. J. Common Mark. Stud.:13517. doi: 10.1111/jcms.13517

[ref61] Schutz R. R. (1951). On the measurement of income inequality. Am. Econ. Rev. 41, 107–122.

[ref62] Seidl C (2007a). “A flat tax with a social component.” Working Paper. Moscow State University—Higher School of Economics.

[ref63] Seidl C. (2007b). “Flat Tax mit Sozialer Grundsicherung: Die Optimale Kombination.” Economics Working Papers 2007–03. Christian-Albrechts-University of Kiel, Department of Economics.

[ref64] Shorrocks A. F. (1980). The class of additively decomposable inequality measures. Econometrica 48, 613–625. doi: 10.2307/1913126

[ref65] Smith A. (1976a) in The Theory of Moral Sentiments. eds. Raphael D. D. Macfie A. L. (Oxford: Clarendon)

[ref66] Smith A. (1976b). An inquiry into the nature and causes of the wealth of nations. Edited and with an introduction, notes, marginal summary, and index by Edwin Cannan; with a new preface by George J. Stigler. Chicago: University of Chicago.

[ref67] Sosu E. M. Schmidt P. (2017). Economic deprivation and its effects on childhood conduct problems: the mediating role of family stress and investment factors. Front. Psychol. 8:1580. doi: 10.3389/fpsyg.2017.01580, PMID: 28955283 PMC5601177

[ref68] Stiglitz J. E. (2011). “Of the 1%, by the 1%, for the 1%.” Vanity Fair, 31 March 2011.

[ref69] Stiglitz J. E. (2015). “When inequality kills.” Project Syndicate.

[ref70] Theil H. (1967). Economics and Information Theory. Amsterdam: North-Holland

[ref71] U.S. National Institute of Standards and Technology (2018). eHandbook of Statistical Methods. Begun in 2003, last updated in 2013. Available at: http://www.itl.nist.gov/div898/handbook/

[ref72] Venkatasubramanian V. (2017). How Much Inequality Is Fair? Mathematical Principles of a Moral, Optimal, and Stable Capitalist Society. New York: Columbia University Press

[ref73] Venkatasubramanian V. Luo Y. Sethuraman J. (2015). How much inequality in income is fair? A microeconomic game theoretic perspective. Phys. A. Statis. Mech. Appl. 435, 120–138. doi: 10.1016/j.physa.2015.04.014

[ref74] Vives JL (1947). Obras Completas. Two Volumes. Translated from Latin into Spanish and with notes and introduction by Lorenzo Riber. Madrid: M. Aguilar

[ref75] Weisskoff R. (1970). Income distributions and economic growth in Puerto Rico, Argentina, and Mexico. Rev. Income Wealth 16, 303–332. doi: 10.1111/j.1475-4991.1970.tb00759.x

